# Gender and Regional Differences in Lung Cancer Mortality in Brazil

**DOI:** 10.31557/APJCP.2020.21.4.919

**Published:** 2020-04

**Authors:** Suellen Nadine de Lima Costa, Fabia Cheyenne Gomes de Morais Fernandes, Camila Alves Dos Santos, Dyego Leandro Bezerra de Souza, Isabelle Ribeiro Barbosa

**Affiliations:** 1 *Undergraduate student in Nursing, Health Science Faculty of Trairi, *; 2 *MSc. Student in the Graduate Program in Collective Health, Federal University of Rio Grande do Norte (UFRN), *; 3 *Federal University of Rio Grande do Norte, Department of Collective Health, Natal, Brazil. *

**Keywords:** Trend, projection, mortality, lung neoplasm

## Abstract

**Background and Objective:**

This was a population-based ecological with data of deaths from the Mortality Information System. The objective of this study was to analyze the temporal trends of mortality induced by bronchi and lung cancer in Brazil and its geographical regions between 2001 and 2015 and secondly to calculate predictions for 2016-2030.

**Material and Methods:**

The mortality trends were analyzed by the Joinpoint regression and calculation of predictions was used the Nordpred software.

**Results:**

There was a reduction trend in lung cancer mortality among Brazilian men living in South and Southeast regions of Brazil. However, there was an increasing trend in lung cancer mortality among Brazilian women living in Northeast, Southeast, and South regions of Brazil. When comparing the last observed period and the last foreseen period for males, it is expected an increase of 12.86% in the number of deaths, justified mainly by the change in population structure, with a reduction in the risk of death by the disease. For women, the expected increase is 26.22%, justified both by population structure, and the increased risk of deaths from the disease. The higher rates will be observed in the southern region of the country, for both sexes.

**Conclusion:**

The mortality induced by lung and bronchial cancer in Brazil was unevenly distributed. However lung cancer incidence had a reducing trend, the mortality caused following it was increased among men. For women, the rates are rising, and until 2030, the mortality load will continue to rise for both.

## Introduction

The bronchial and lung cancer is the most common neoplasm that is associated with high mortality, posing higher frequency among men. In 2012, 1.82 million people were diagnosed with lung cancer, out of whom, 1.6 million patients died of this disease in the world. This amount corresponds to 19.51% of all cancer deaths. The highest incidence of lung cancer was reported among men in Central and Eastern Europe, but among women in North America and Northern Europe (Ferlay et al., 2015).

Regarding the distribution of the disease around the world, countries with the highest Human Development Index (HDI) are those that present higher rates of lung cancer incidence and lung cancer-induced mortality (Wong et al, 2017). With respect to ethnicity, a higher incidence is reported in black people (Schabath et al., 2016). In Brazil, corresponds to 8.7% of the cancers diagnosed in men (the second most incident) (Inca, 2018). 

Regarding causal factors, the disease is directly related to tobacco smoking. However, there are other predisposing factors, such as air pollution, inadequate diet, alcohol consumption, mutation, and single nucleotide polymorphism (Akhtar and Bansal, 2017). Factors such as exposure to radon, indoor coal burning, and genetic susceptibility can also contribute to incidence of this disease (Pallis and Syrigos, 2013). 

Thus, the epidemiological understanding of how lung and bronchial cancer behave and investigating its geographical distribution and prevalence over time is essential to develop strategies and plans to more susceptible groups. The objective of this study was to analyze the temporal trend in bronchial and lung cancer-induced mortality in Brazil from 2001 to 2015 and to predict the mortality rate predictions for the period of 2016-2030.

## Materials and Methods

An ecological study of temporal series based on secondary data recorded in the Mortality Information System (SIM) of the Department of Informatics of the Brazilian Unified Health System (DATASUS). We analyzed deaths caused from malignant neoplasm of bronchus and lungs (C34) categorized from the International Statistical Classification of Diseases (ICD) and Related Health Problems- 10ª Review (ICD-10), occurred in Brazil from 2001 to 2015. These data were designed by the year 2030 and. The data were analyzed according to sex and age group.

Although SIM in Brazil has gotten a significant gain in quality in recent years, the use of secondary data on mortality is subject the sub-enrollment. Consequently, to correct the sub-enrollment, was used a sequence of procedures for creating a correction factor, from the information of Redistribution for Chapters of Deaths corrected by Active Search (Brasil, 2015), an initiative by the Brazilian Ministry of Health, available on the site of the DATASUS. 

The correction factor was calculated for each age group, period, region, and sex based on the percentage difference between the number of deaths reported to SIM and that of redistributed deaths, based on Chapter II (Neoplasms) of the ICD-10. The referred difference was expressed in decimal values and the value 1 corresponded to a change of 100% according to the formula proposed by Santos and Souza (2018):

D= (NR-NS)/NS

In which:

D = Ratio between the number of deaths redistributed and registered to SIM in relation to deaths registered to SIM by neoplasms

NR = Number of deaths redistributed by neoplasms, and 

NS = Number of registered deaths to SIM by neoplasms.

This obtained difference was added to the value 1 (neutral factor in a multiplication) for calculation of correction factor in accordance to the following formula:

F= 1 + D

In which:

F = Factor of correction of chapter II (neoplasms), and

D = Ratio between the number of deaths redistributed and registered to SIM in relation to deaths registered to SIM by neoplasms.

This factor was multiplied by the number of deaths induced by bronchial and lung cancer, assuming the correction factor for neoplasms is applicable to this cancer. The formula is described below:

OC= F x NOS

OC = Number of deaths induced by bronchus and lung cancer corrected ,

NOS = Number of registered deaths to SIM for bronchus and lung cancer, and

F = Factor of correction of neoplasms.

Considering the information on the number of deaths, the standardized mortality rates were calculated and adjusted according to world population for every 100,000 inhabitants. The population data by region, sex, and age were obtained from the Demographic census and from the intercensal estimate, on the Brazilian Institute of Geography and Statistics’s website (IBGE).

Mortality predictions were calculated by the year 2030 per five-year periods, using the age-period-cohort model (APC) and Nordpred software (Cancer Registry of Norway, Oslo, Norway) inscribed in the R statistical software.

The results on the predictions were presented for the total deaths observed and expected for each period for Brazil and its regions . For each period, mortality rates were calculated based on the adjusted population world standard for global comparisons, expressed for 100,000 inhabitants per year (ASW∕100,000 inh) (Doll et al., 1966).

Mortality trends from 2001 to 2015 were analyzed using Joinpoint regression and Joinpoint Regression software (version 4.4.0). The goal of the analysis was to identify the occurrence of possible points in which there was a significant change in trend.

The method is based on the model with a maximum of 3 change points. The final selected model was the model more adjusted with the Annual Percentage Change (APC) based on the trend of each segment, estimating if these values were statistically significant at p<0.05 or not. The used significance tests were based on the permutation method of Monte Carlo and in the calculation of the annual percentage variation of ratio logarithm (Kim et al., 2000).

In the description of trends, the terms “increase” and “reduction” meaned that the trend was statistically significant. 

The annual changes in the number of deaths in the last projected period (2026-2030) was also calculated compared to the last observed period (2011-2015), evaluating whether the proportion of this change had resulted from a modification in the risk of dying from bronchi and lung cancer or due to demographic changes (population size or structure). These two components can be non-zero and present a positive or negative direction. The calculation can be expressed as follows (Møller et al., 2003):

Δ_tot_ = Δ_risk_ + Δ_pop_= (N_fff_ – N_off_) + (N_off_ – N_ooo_)

Δ_tot_ is the total change, Δ_risk_ is the change in the risk function, Δ_pop_ is the change in population, N_ooo_ is the number of cases observed, N_fff_ is the number of cases projected, and N_off_ is the number of expected cases when mortality rates increase during the observed period.

## Results

**Figure 1:**
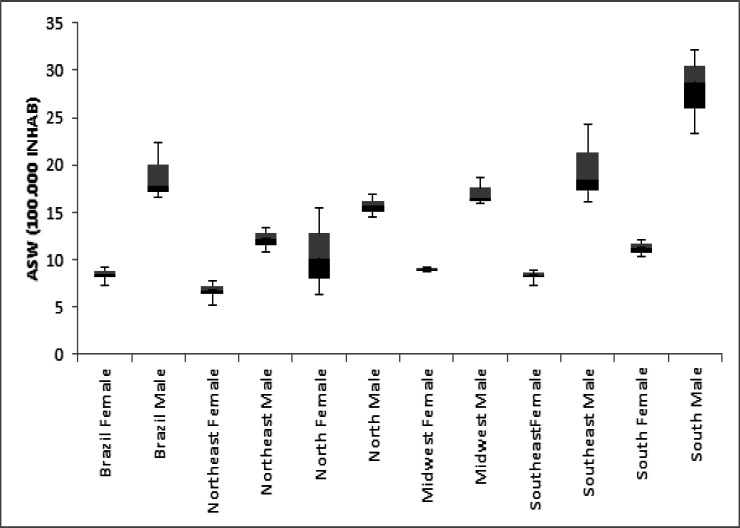
Standardized Mortality Rates of Lung Cancer in Brazil and Its Geographies Regions, According to Sex for the Period of 2001-2015

From 2001 to 2015, there were 351.815 deaths caused by malignant neoplasm of bronchus and lung cancer in Brazil (63.67% affecting males). The standardized mortality rate for the world’s population for women in Brazil ranged from 7.28 deaths/100,000 inhabitants in 2001 to 9.30 in 2015. For men, this rate varied from 19.70 in 2001 to 16.63 in 2015. The highest mortality rates were recorded for men in the South and Southeast regions, but for women in North and South (Figure 1). Regarding the ratio for deaths between women and men for the period 2001-2015, there was an average of 1:1.7. 

In the analysis of the historical series of mortality rates for males, there was a general trend to reduction of mortality for Brazil, with the occurrence of two Joinpoints, one in the year 2005, with increase followed by reduction; and a second joinpoint in 2008, with a less pronounced reduction. This pattern was similar to the Southeast and South. However, the Northeast region showed a trend to increase, followed by a period of reduction and a new increase. North and Midwest showed stability.

The analysis of trends in female population showed increased rates for Brazil with the occurrence of two joinpoints, in 2005 and 2008, separating a period of increase, followed by stability and subsequent increase. The pattern was similar to the Northeast and Southeast. The Northern region was the only that presented reduction for women, while the data of the Midwest showed stability, both without joinpoints (Table 1).

Tables 2 and 3 presents the number of deaths and mortality rates for the periods observed and projected for female and male, respectively. Analyzing the data for Brazil in the five-year-period 2026-2030, it was projected the occurrence of 99.377 female deaths by lung cancer, while for males this number was 105.696 deaths. Mortality rates for females will increases in the future, especially on Northeast, Midwest, and South. For males, reductions will occur, mainly in South, Southeast, Center-West and North.

**Figure 2:**
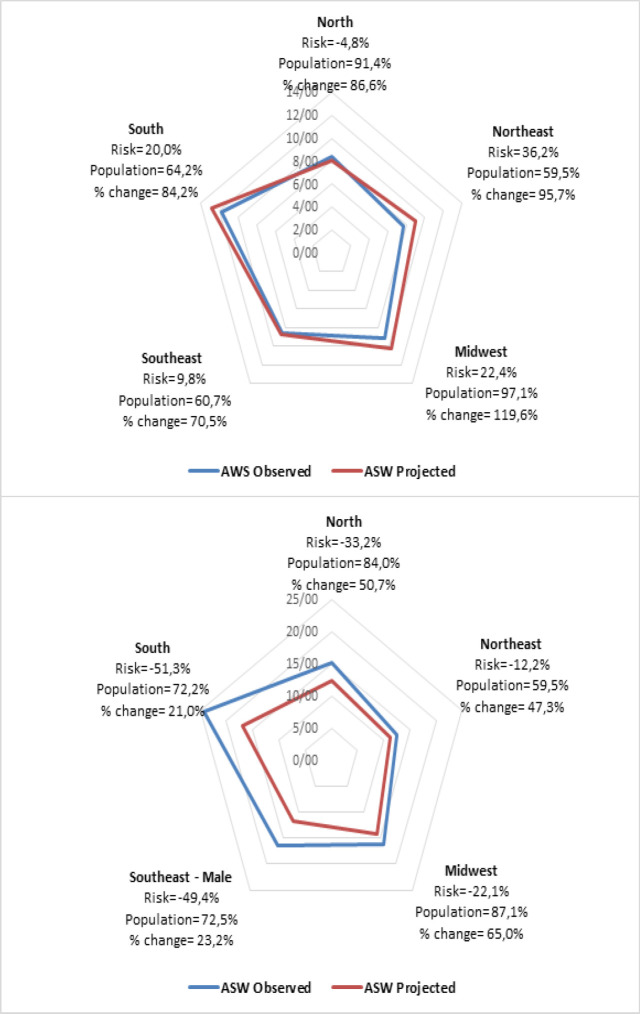
Age-standardized Rates (ASW), Total Change (change), Relative Change Due to Risk (risk) and Modified Population (population), being Female and male, between 2011-2015 (observed) and 2026-2030 (predicted) of Lung Cancer Mortality in Brazil

Figure 2 presents the mortality rates for lung cancer, observed and projected periods, according to the influence of the risks and the population structure of Brazil and regions. For Brazil, change to mortality in females, is explained mainly by the change in the brazilian demographic structure. Also, the Midwest and Northeast regions stand out for which there will be an increase in the mortality rate of the projected period compared to the observed, with a high percentage of change and the positive influences of the demographic structures overlapping the risk of dying by the disease. In males, the percentage of change is justified mainly by the change in the demographic structure brazilian, accompanied by a reduction in the risk of dying by the disease. 

**Table 1:** Temporal Trends for Lung Cancer Mortality in Brazil and Its Regions: Number of Deaths, APC, Confidence Interval, and Year of the Joinpoint

	Number of deaths	APC1 (CI 95%)	Joinpoint	APC2 (CI 95%)	Joinpoint	APC 3
Females						
Brazil	127,805	5.1* (3.6;-6.7)	2005	-2.8 (-7.3;1.9)	2008	1.9* (1.2;2.5)
Northeast	26,381	7.6* (4.9;10.3)	2005	-3.0 (-10.4;5.0)	2008	3.8* (2.8;4.9)
North	6,178	-6.4* (-11.5;-1.0)				
Midwest	7,799	0.4 (-0.7;1.6)				
Southeast	59,520	5.2* (3.8;6.6)	2005	-3.0 (-7.0;1.1)	2008	1.4* (0.8;2.0)
South	27,211	1.1* (0.6;1.7)				
Male						
Brazil	224,010	1.9* (0.3;3.6)	2005	-5.8* (-10.6;-0.9)	2008	-0.9* (-1.6;-0.2)
Northeast	38,910	5.7* (3.2;8.3)	2005	-3.2* (-4.8;-1.5)	2011	5.1* (2.6;7.7)
North	11,215	-1.1 (-2.2;0.1)				
Midwest	13,269	- 0.8 (-1.7;0.2)				
Southeast	104,698	1.6 (-2.9;6.4)	2004	-4.8* (-9.1;-0.3)	2008	-1.8* (-3.0;-0.6)
South	54,146	3.0 (-2.5;8.8)	2004	-2.9* (-3.6;-2.2)		

95% CI, 95% confidence intervals; APC,annual percentage changes; Statistically significant p<0.05.

**Table 2:** Mortality Induced by Lung Cancer in Brazil and Its Regions among Women: The Number of Observed and Projected Deaths by Age and Mortality Rates Adjusted to the World Population (ASW/100,000 inhabitants)

	Observed			Projected		
	2001-2005	2006-2010	2011-2015	2016-2020	2021-2025	2026-2030
BRAZIL						
Age (years)						
0-39	1,017	1,064	1,088	1,101	1,063	1,023
40-64	14,978	18,854	23,048	27,037	29,555	30,126
≥ 65	17,495	23,010	30,444	40,206	52,864	68,228
ASW	7.62	8.3	8.89	9.35	9.61	9.61
Northeast						
Age (years)						
0-39	323	284	316	321	318	305
40-64	2,860	3,593	4,649	5,699	6,315	6,665
≥ 65	3,344	4,543	6,447	8,943	12,035	15,359
ASW	5.86	6.71	7.65	8.51	8.95	8.96
North						
Age (years)						
0-39	106	109	99	121	132	138
40-64	760	822	994	1,170	1,301	1,379
≥ 65	842	1,026	1,414	1,873	2,466	3,162
ASW	8.46	8.01	8.36	8.44	8.34	8.02
Midwest						
Age (years)						
0-39	69	75	90	107	121	131
40-64	792	1,055	1,395	1,819	2,249	2,611
≥ 65	1,011	1,377	1,933	2,664	3,592	4,763
ASW	8.2	8.65	9.22	9.75	10.11	10.31
Southeast						
Age (years)						
0-39	392	434	421	421	416	440
40-64	6,475	8,391	10,148	11,594	12,127	11,684
≥ 65	8,392	10,969	13,936	17,810	23,117	29,664
ASW	7.51	8.21	8.55	8.78	8.87	8.77
South						
Age (years)						
0-39	141	157	160	168	162	154
40-64	2,930	3,790	4,661	5,514	6,120	6,295
≥ 65	3,728	5,000	6,636	8,751	11,444	14,653
ASW	9.99	11.01	11.68	12.29	12.69	12.82

**Table 3:** Mortality Induced by Lung Cancer in Brazil and Its Regions among Men: The Number of Observed and Projected Deaths by Age and Mortality Rates Adjusted to the World Population (ASW/100.000 inhabitants)

	Observed			Projected		
	2001-2005	2006-2010	2011-2015	2016-2020	2021-2025	2026-2030
BRAZIL						
Age (years)						
0-39	1,429	1,395	1,451	1,522	1,650	1,813
40-64	28,136	29,775	30,441	31,167	30,946	30,398
≥ 65	38,468	43,389	49,709	55,735	63,809	73,485
ASW	19.72	18.35	16.79	15.14	13.74	12.62
Northeast						
Age (years)						
0-39	512	474	491	493	495	498
40-64	4,623	4,999	5,447	5,851	6,090	6,145
≥ 65	6,199	7,218	8,917	10,846	12,960	15,242
ASW	12.19	12.19	12.44	12.36	11.93	11.27
North						
Age (years)						
0-39	146	187	178	198	202	199
40-64	1,384	1,437	1,567	1,725	1,931	2,207
≥ 65	1,750	2,019	2,541	2,990	3,497	4,055
ASW	16.83	15.69	15.69	14.17	13.19	12.36
Midwest						
Age (years)						
0-39	90	97	120	145	171	184
40-64	1,556	1,780	1,997	2,227	2,405	2,533
≥ 65	1,987	2,464	3,161	3,946	4,889	5,991
ASW	17.06	16.61	16.23	15.63	14.91	14.17
Southeast						
Age (years)						
0-39	547	481	501	552	627	750
40-64	13,457	13,932	13,965	14,021	13,524	12,920
≥ 65	18,606	20,501	22,792	24,859	28,123	32,214
ASW	20.75	18.62	16.47	14.43	12.87	11.76
South						
Age (years)						
0-39	179	191	185	157	142	131
40-64	6,850	7,426	7,328	7,167	6,876	6,550
≥ 65	9,193	10,821	12,030	13,141	14,799	16,962
ASW	29.6	27.93	24.34	21.01	18.6	17

## Discussion

In the world, the bronchial and lung cancer is a tumor associated with high mortality (Islami et al., 2015; Mao et al., 2016; Hirsch et al., 2017) and diagnosis in later stages (Hirsch et al., 2017). About 1.8 million people are diagnosed annually by this cancer, accounting for 1.6 million deaths and a five-survival rate of 4% to 17% (Hirsch et al., 2017; Ziebarth, 2018).

The present study evaluated lung cancer mortality in Brazil from 2001 to 2030. The rate of lung cancer mortality among men was ranged from 19.72 (2001-2005) to 12.62 (2026-2030), denoting fee reductions, while for females, it was ranged from 7.62 (2001-2005) to 9.61 (2026-2030), Indicating rising trend.

Regarding different regions of the country, South region had the highest lung cancer-induced mortality both for men and women. For men, the Southeast region ranked the second position regarding mortality rates for the observed period followed by the Midwest region. This region; however, overcomes the Southeast for the period projected. North overcomes Northeast, which has the lowest rates among men. The higher rates of lung cancer-induced mortality among women were detected in the South, followed by Midwest and Southeast. North, in turn, has higher rates than Northeast in the period observed, whose pattern reverses for the projected period. 

The result of this study was compatible with those reported in the literature. During 1996-2011, a study was done on the age group of 30-69 years old to assess the trends in lung cancer mortality. In the aforementioned study, a reduction trend was identified among men and but an increase trend among women; however, higher rates of lung cancer-induced mortality was detected among men (Malta et al, 2016).

About mortality data, study with global estimates mortality for the year 2012 shows that was found a rate of 47.6 deaths/100,000 inhabitants among men for the Central and Eastern Europe and 44.8 to Asia Oriental. Among women, rates are 23.5 in North America and 19.1 in Northern Europe. In sub-Saharan Africa, the rates are usually lower, varying from 4.4 (men) to 2.2 (women) (Islami et al, 2015). According to World Health Organization (WHO), in general, there are stable rates for women aged 30-49 years old, and most countries showed increased rates among older women (50-74 years) (Torre et al., 2014).

In Europe, several studies were conducted to study lung cancer. Some studies showed decreasing trend of lung cancer incidence among men (Cayuela et al., 2012; Malvezzi et al., 2013; Martín-Sánchez et al., 2016; Malvezzi et al., 2017), but its increasing trend among women over the last few years (Cayuela et al., 2012; Bosetti et al., 2012; Martín-Sánchez et al., 2016; Malvezzi et al., 2017). This increase trend was occurred in Spain mainly after 1997 (Martín-Sánchez et al., 2016), but also in predictions performed in France (Eilstein and Eshai, 2012). The present study showed a higher number of deaths among men a reduction trend in lung cancer incidence among men over the period under study, which was in line with results reported studies done in Spain (Cayuela et al., 2012) and Sweden (Abdoli et al., 2014).

One study in African evaluated the trend of lung cancer mortality between 1995 and 2006 and found a similar result to what reported in Europe, revealing a decline among men and an increase among women (Bello et al., 2011). In Asia, a number of studies were performed in Japan, an increase in lung cancer mortality among men and women was identified, while inconsistent results were reported from other countries about the trend of lung cancer mortality among men (Funatogawa et al., 2013). In China, increased mortality rates were identified in the last 3 decades, mainly among rural residents (Wang et al., 2016). 

Regarding risk factors of lung cancer, most studies identified tobacco smoking as the main risk factor (Eilstein and Eshai, 2012; Islami et al., 2015; Martín-Sánchez et al., 2016; Torre et al., 2016; Malvezzi et al., 2017). Based on WHO estimates, tobacco consumption will lead to death of about 10 million people per year by 2025, out of which 30% is induced by lung cancer. Currently, lung cancer is the second leading cause of mortality in the world (Mao et al., 2016).

The higher rate of lung cancer among men can be attributed to their historical habit of smoking (Malvezzi et al., 2017), that has been expanded during the Industrial Revolution (Filho et al., 2010). In the world, the highest prevalence of smoking among men was observed in East and Southeast Asia, as well as Eastern Europe. For women, the consumption is more worrying in European countries, followed by Oceania, and in North and South Americ (Islami et al., 2015).

Another important finding of this study was related to the increased rate of among women in all regions of Brazil during the evaluated period, except for the Midwest. It is plausible to associate the increase in cigarette consumption, that becomes similar between genders over time (Borsoi et al., 2011) to that elevation occurred in mortality for lung cancer (Filho et al., 2010; Alberg et al., 2013; Malvezzi et al., 2013; Islami et al., 2015; Martín-Sánch et al., 2016). Therefore, smoking habit is disturbing not only in developing countries (Alberg et al, 2013), as China (Marx, 2012), but also in developed countries, such as Japan, which was verified the reduction of the age of onset of smoking (Funatogawa et al., 2013). The consumption, however, shows an uneven distribution among populations of different social classes, more frequently in those with lower levels of schooling and low income (Filho et al., 2010; Wong et al., 2017).

In Brazil, the highest rate was found in the South of the country. This region has is the biggest cigarette producer in the country, particularly Rio Grande do Sul (50% of the national production of tobacco), which can be related to increased cigarette consumption based on data reported by IBGE and INCA pointing higher prevalence of smoking in South (Filho et al., 2010).

Regarding the cases of the disease in non-smokers, the highest recorded amount is in women due to passive tobacco consumption (Filho et al., 2010; Couraud et al., 2012), while exposure to environmental carcinogenic occupational agents is more common in men (Alberg et al., 2013). It is also possible associate with hormonal factors, hereditary components, infections or respiratory diseases, air pollution, lifestyle and unhealthy habits, and exposure to ionizing radiation, especially radon (Couraud et al., 2012; Mao et al., 2016; Akhtar et al., 2017), arsenic, and asbestos and (Mao et al., 2016; Wang, 2016; Akhtar et al., 2017). In addition, HIV infection and Epstein-barr virus (Akhtar et al., 2017) can be named as other risk factors of this malignancy.

The growth in the prevalence of lung cancer-induced mortality among Brazilian women implies a need for preventive and monitoring measures against smoking (Alberg et al, 2013; Wang et al, 2016; Torre et al, 2016; Mao et al, 2016; Ziebarth, 2018), conserving the environment, improving life habits (Wang et al, 2016; Torre et al, 2016), doing vaccination, and diagnosing at early stages (Torre et al, 2016).

Considering the limitations of this study, it is possible to mention the use of secondary data, likely to underreporting. Thus, some discrepancies are possible in the rates of lung cancer due to the differences in the records and data quality (Rafiemanesh et al., 2016). Another limitation was related to the consideration of the correction factor for lung cancer is similar to that of neoplasms in general. This fact, however, was minimized by separate correction by gender and by age groups for all regions and periods. 

In conclusion, the study allowed the identification of the most critical regions for the occurrence of Lung Cancer in the coming years, especially in the South, which identifies the highest rates of Brazil. Furthermore, it is valid to note the increase in rates among women, a result consistent with the world literature, and that implies the need for preventive measures and public policies for tobacco control, which is the main factor of risk for this type of cancer. 

## Highlights

• There was a reduction trend in lung cancer mortality among Brazilian men.

• There was an increasing trend in lung cancer mortality among Brazilian women.

• The higher rates of was observed in the southern region of the country for both sexes.
